# Temporal trends of antithrombotic therapy for stroke prevention in Korean patients with non-valvular atrial fibrillation in the era of non-vitamin K antagonist oral anticoagulants: A nationwide population-based study

**DOI:** 10.1371/journal.pone.0189495

**Published:** 2017-12-20

**Authors:** So-Ryoung Lee, Eue-Keun Choi, Kyung-Do Han, Myung-Jin Cha, Seil Oh, Gregory Y. H. Lip

**Affiliations:** 1 Department of Internal Medicine, Soon Chun Hyang University Hospital Seoul, Seoul, Republic of Korea; 2 Department of Internal Medicine, Seoul National University Hospital, Seoul, Republic of Korea; 3 Department of Biostatistics, College of Medicine, The Catholic University of Korea, Seoul, Republic of Korea; 4 Institute of Cardiovascular Sciences, University of Birmingham, United Kingdom; 5 Aalborg Thrombosis Research Unit, Department of Clinical Medicine, Aalborg University, Aalborg, Denmark; Monash University, AUSTRALIA

## Abstract

**Background:**

Following their introduction, the non-vitamin K antagonist oral anticoagulants (NOACs) are increasingly prescribed in Asia for stroke prevention in patients with non-valvular atrial fibrillation (AF). Few contemporary data are available on temporal trends in antithrombotic therapy use in Asian countries, in the era of NOACs.

**Methods and results:**

Using the National Health Insurance Service database of the entire Korean adult AF population, the use of aspirin, vitamin K antagonist, and NOACs between 2008 and 2015 were analyzed (n = 276,246 in 2015). Most of the included cohort had CHA_2_DS_2_-VASc score ≥ 2 (78.2% in 2008 and 83.2% in 2015), yet approximately 17% were prescribed no antithrombotic therapy throughout the study period. Aspirin prescription consistently decreased (from 48.2% to 31.5%) over time, while OAC prescription significantly increased from 34.7% to 50.6%. NOAC prescriptions accounted for 50% of total OAC prescription in 2015. Similar trends in antithrombotic therapy were found both in men and in women, but women were more likely to be undertreated with OAC. Female gender, presence of vascular disease and prior intracranial hemorrhage were associated with OAC underuse.

**Conclusions:**

Between 2008 and 2015, a greater proportion of AF patients received OAC treatment with increasing NOAC prescription trends in the recent 3 years. A substantial proportion (approx. 50%) of Korean patients with AF still remain undertreated.

## Introduction

Atrial fibrillation (AF) is associated with an up to 5-fold increase in the risk of stroke, and thus stroke prevention is fundamental in the management of patients with AF [1]. For decades, vitamin K antagonists (VKAs) (e.g., warfarin) were the only available oral anticoagulants (OACs) for these patients. A meta-analysis of the historical randomized trials showed that VKAs effectively reduced the risk of stroke by 64% and mortality by 26% compared to placebo or control [2]. Despite the clear evidence of benefits, VKAs are underused in a large number of patients with AF who need OACs, for several reasons [3–5]. The narrow therapeutic range and the need for frequent monitoring and dose adjustments, as well as diet and drug interactions made clinicians and patients reluctant to use VKAs [3].

Asian countries are known to have lower prevalence and incidence of AF than Western countries, but its rate is rapidly growing [6]. In recent years, the landscape of stroke prevention in patients with AF changed with the availability of the non-VKA oral anticoagulants (NOACs) [7–9]. These drugs offer better efficacy, safety and convenience compared to VKA [1]. Furthermore, changes in treatment guidelines promoting NOAC use and discouraging aspirin have increased focus on OAC use for stroke prevention in AF [7,10,11]. Despite these major changes in stroke prevention in AF, few contemporary data are available about the changes of antithrombotic prescribing patterns, especially in the Asian population [12–14].

In this study, our aim was to describe temporal changes and the current state of antithrombotic therapy use in an Asian country, in the era of NOACs. Second, we evaluated the differences in antithrombotic therapy according to patients’ baseline characteristics.

## Methods

### Data sources and study patients

We used the data from the national health claims database from the National Health Insurance Service (NHIS) of Korea [15], a mandatory universal health insurance program that provides comprehensive medical care coverage to 97% of the entire Korean population (approx. 50 million people). The Medical Aid program covers the remaining 3% of the population, the low-income population. Since 2006, the information from the Medical Aid program has been integrated within a single NHIS database. Therefore, the NHIS claims database truly includes the claims data of the entire Korean population. The NHIS database consists of diagnoses, procedures, prescription records, and demographic information. We identified diagnoses using the International Classification of Disease, Tenth Revision, Clinical Modification (ICD-10-CM) codes. Data for all Koreans aged ≥ 20 years from January 1, 2008, to December 31, 2015, were included. This study was exempt from review by the Seoul National University Hospital Institutional Review Board (1607-056-775).

### Definition of non-valvular AF

Prevalent AF was identified using the ICD-10-CM codes (I480–I484, and I489). To improve the diagnostic accuracy and avoid overestimation due to inclusion of subjects with transient AF, we included only patients with AF with ≥ 1 diagnoses during hospitalization or ≥ 2 diagnoses in outpatient clinic [6,16–18]. To limit the study population to non-valvular AF, patients with mitral stenosis (I50, I52, and I59) or mechanical heart valves (Z952–Z954) were excluded.

### Comorbidities

The definitions of patients’ comorbidities are summarized in Supplemental Table 1. We identified the presence of comorbidities as concurrent diagnoses from 1 year ago each patient to be diagnosed as AF. Hypertension was defined based on a combination of diagnostic codes and the use of ≥1 anti-hypertensive drug. Diabetes mellitus was defined based on diabetes diagnostic codes and at least 1 anti-diabetic agent prescription. Heart failure, prior stroke/transient ischemic attack (TIA)/systemic thromboembolism (TE), prior myocardial infarction (MI), peripheral artery disease (PAD), and prior intracranial hemorrhage (ICH) were also defined using ICD-10-CM codes. The definition of these comorbidities has been validated in previous studies based on the NHIS cohort [6,17–21].

**Table 1:** Baseline characteristics of patients with CHA_2_DS_2_-VASc score ≥ 2 in 2015 (n = 230,332).

	No OAC, total (n = 113,851)	No therapy (n = 41,375)	Aspirin (n = 72,476)	OAC, total (n = 116,481)	VKA (n = 57,950)	NOAC (n = 58,531)
Age (mean ± SD)	72.1±11.2	72.3±11.8	72.0±10.8	72.4±9.8	71.7±10.3	73.1±9.1
Age 65–74, n (%)	36,891 (32.4)	12,455 (30.1)	24,436 (33.7)	41,305 (35.5)	20,190 (34.8)	21,115 (36.1)
Age ≥ 75 years, n (%)	52,087 (45.8)	19,837 (47.9)	32,250 (44.5)	54,017 (46.4)	25,645 (44.3)	28,372 (48.5)
Female, n (%)	57,479 (50.9)	23,177 (56.0)	34,302 (47.3)	53,036 (45.5)	25,633 (44.2)	27,403 (46.8)
Hypertension, n (%)	82,251 (72.2)	26,875 (64.9)	55,376 (76.4)	87,257 (74.9)	42,976 (74.2)	44,281 (75.7)
Diabetes mellitus, n (%)	29,801 (26.2)	10,097 (24.4)	19,704 (21.2)	33,861 (29.1)	16,764 (28.9)	17,097 (29.2)
Heart failure, n (%)	43,441 (38.2)	13,881 (33.6)	29,560 (40.8)	53,287 (45.8)	26,570 (45.8)	26,717 (45.7)
Prior stroke/TIA/TE, n (%)	24,579 (21.6)	9,617 (23.2)	14,962 (20.6)	47,852 (41.1)	21,393 (36.9)	26,459 (45.2)
Stroke, n (%)	18.993 (16.7)	7,907 (19.1)	11,086 (15.3)	40,868 (35.1)	17,776 (30.7)	23,092 (39.5)
TIA, n (%)	3,498 (3.1)	1,103 (2.7)	2,395 (3.3)	4,330 (3.7)	1,750 (3.0)	2,580 (4.4)
TE, n (%)	4,037 (3.6)	1,206 (2.9)	2,831 (3.9)	7,616 (6.5)	3,778 (6.5)	3,838 (6.6)
Vascular disease, n (%)	30,225 (26.5)	10,560 (25.5)	19,665 (27.1)	20,855 (17.9)	10,008 (17.3)	10,847 (18.5)
Prior MI, n (%)	6,952 (6.1)	1,861 (4.5)	5,091 (7.0)	5,352 (4.6)	2,662 (4.6)	2,690 (4.6)
PAD, n (%)	24,786 (21.8)	9,089 (21.9)	15,697 (21.7)	16,429 (14.1)	7,800 (13.5)	8,629 (14.7)
Prior ICH	3,146 (2.8)	1,511 (3.6)	1,635 (2.3)	3,250 (2.8)	1,436 (2.5)	1,814 (3.1)
CHA_2_DS_2_-VASc score (mean ± SD)	3.8±1.5	3.7±1.5	3.8±1.5	4.2±1.5	4.0±1.5	4.3±1.5
CHA_2_DS_2_-VASc score, n (%)						
Score = 2	27,682 (24.3)	10,140 (24.5)	17,542 (24.2)	18,380 (15.8)	10,810 (18.7)	7,570 (12.9)
Score = 3	27,921 (24.5)	10,410 (25.2)	17,511 (24.2)	25,329 (21.8)	13,225 (22.8)	12,104 (20.7)
Score = 4	24,913 (21.9)	9,033 (21.8)	15,880 (21.9)	26,993 (23.2)	13,360 (23.1)	13,633 (23.3)
Score = 5	18,217 (16.0)	6,685 (16.2)	11,532 (15.9)	22,481(19.3)	10,500(18.1)	11,981 (20.5)
Score = 6	9,368 (8.2)	3,271(7.91)	6097(8.41)	14067(12.08)	6243(10.77)	7824(13.37)
Score = 7	4,177 (3.7)	1,382(3.34)	2795(3.86)	6800(5.84)	2823(4.87)	3977(6.79)
Score = 8	1,349 (1.2)	388(0.94)	961(1.33)	2106(1.81)	857(1.48)	1249(2.13)
Score = 9	224 (0.2)	66(0.16)	158(0.22)	325(0.28)	132(0.23)	193(0.33)

Abbreviation: ICH, intracranial hemorrhage; MI, myocardial infarction; NOAC, non-vitamin K antagonist oral anticoagulants; OAC, oral anticoagulants; PAD, peripheral artery disease; SD, standard deviation; TE, thromboembolism, TIA, transient ischemic attack.

### Assessment of stroke risk

According to the current guidelines, the stroke risk of patients with AF was estimated based on the CHA_2_DS_2_-VASc score [10,11,22,23]. In the most recently updated European guidelines, OAC therapy is recommend for all male AF patients with a CHA_2_DS_2_-VASc score of 2 or more and all females with CHA_2_DS_2_-VASc score of 3 or more [11]. Also, in male AF patients with a CHA_2_DS_2_-VASc score of 1 and females with a CHA_2_DS_2_-VASc of 2, OAC should be considered considering individual characteristics and patient preferences [11]. We calculated each patient’s CHA_2_DS_2_-VASc score by assigning 1 point for age between 65 and 74 years, female sex, and the presence of hypertension, diabetes mellitus, heart failure, and vascular disease (prior MI or presence of PAD), and adding 2 points for a prior stroke/TIA/TE or age of ≥ 75 years [22].

### Antithrombotic therapy among study population

We reviewed the inpatient and outpatient prescription records of each patient to determine if there was any prescription of antithrombotic therapy including aspirin, VKAs, and NOACs. Patients were categorized into four treatment groups: no therapy, aspirin only, VKA ± aspirin as the VKA group, and NOAC ± aspirin as the NOAC group. In Korea, dabigatran was introduced in 2011, rivaroxaban in 2012, and apixaban in 2013. Edoxaban was introduced in Korea in 2016, and thus, is not part of this analysis. There are special considerations about the medical insurance imbursement policy of NOAC prescription for stroke prevention in patients with AF. In 2013, NOAC use for stroke prevention in patients with AF was approved only for patients with VKA failure, such as critical bleeding events or labile international normalized ratio (INR) values, and CHA_2_DS_2_-VASc ≥ 2. In 2015, reflecting the updated results of randomized controlled trials for NOACs and Korean AF management guidelines on antithrombotic therapy in patients with non-valvular AF, NOAC use in Korea was more widely approved in patients with AF and a CHA_2_DS_2_-VASc score ≥ 2 without any conditional terms [7,8,10,23].

### Statistical analysis

Data management and statistical analyses were performed using SAS, version 9.3 (SAS Institute, Cary, NC, USA) and SPSS statistical package, version 19.0 (SPSS Inc., Chicago, IL, USA). Descriptive statistics of the proportion of antithrombotic therapy prescription were examined. Categorical variables were presented as numbers and percentages. The chi-square test was performed to evaluate the differences between categorical variables. Continuous data were described as medians with interquartile ranges or means with standard deviations. Comparison of continuous variables was performed using the independent t-test. Statistical significance was set at two-tailed p values < 0.05.

The distribution of antithrombotic therapy was described in relation to the total study population. We stratified patients according to their stroke risk using CHA_2_DS_2_-VASc score, and described temporal changes in prescription trends and gender differences. We also analyzed differences of baseline characteristics among patients with no antithrombotic therapy, aspirin, and OACs (VKAs vs. NOACs) in the 2015 population with CHA_2_DS_2_-VASc score ≥ 2. We used the Cochran-Armitage trend test for evaluation of temporal trends in OAC prescription over time. To evaluate the factors associated with OAC underuse, univariable and multivariable binary logistic regression analysis was performed. Odds ratios (OR) with 95% confidence intervals (CI) were calculated as an estimated risk associated with OAC underuse.

## Results

### Baseline characteristics of study population

The study population has been recently described by our group [6]. The total number of patients with non-valvular AF increased gradually and consistently throughout the study period from 150,301 in 2008 to 276,246 in 2015. The mean CHA_2_DS_2_-VASc score was 3.3±1.9 in 2008 and 3.4±1.9 in 2015, with the majority (78.2% in 2008 and 83.2% in 2015) of patients falling into a high stroke risk category (CHA_2_DS_2_-VASc score ≥ 2, Table 1). Thus, the proportion of patients with AF who were strongly recommended for OAC treatment for stroke prevention has increased throughout the study period (p for trend < 0.001) [6].

### Temporal trends in antithrombotic therapy between 2008 and 2015

#### Total study population

Among the total study population, 21.9% in 2008 and 21.2% in 2015 received no antithrombotic therapy, with the proportion taking no antithrombotic therapy being constant, with no statistically significant temporal trends of change throughout the study period (Panel A in S1 Fig). In 2008, AF patients were prescribed aspirin more frequently than OACs (46.2% vs. 32.0%), with this proportion reversed in 2015, when OAC therapy was prescribed in 46.0% of patients, and aspirin in 32.8%. OAC prescription showed a constant increase from 32.0% in 2008 to 46.0% in 2015, and aspirin prescription decreased from 46.2% in 2008 to 32.8% in 2015 (p for trend < 0.001).

Between 2008 and 2012, when NOACs were not approved yet, all the patients who needed OACs were prescribed VKAs. After limited approval of NOACs in 2013, 34.7% were prescribed VKAs and 4.2% NOACs. After wider approval in 2015, among 46.0% of patients taking OACs, nearly half (22.2%) received NOACs, while 23.8% received VKAs. Among patients on NOACs, 43.3% received rivaroxaban, 34.2% received dabigatran and 22.5% received apixaban in 2015 (Panel B in S1 Fig). Temporal trends in antithrombotic therapy for the total study population are shown in Panel A in S1 Fig.

#### High stroke risk patients (CHA_2_DS_2_-VASc score ≥ 2)

**Fig 1:**
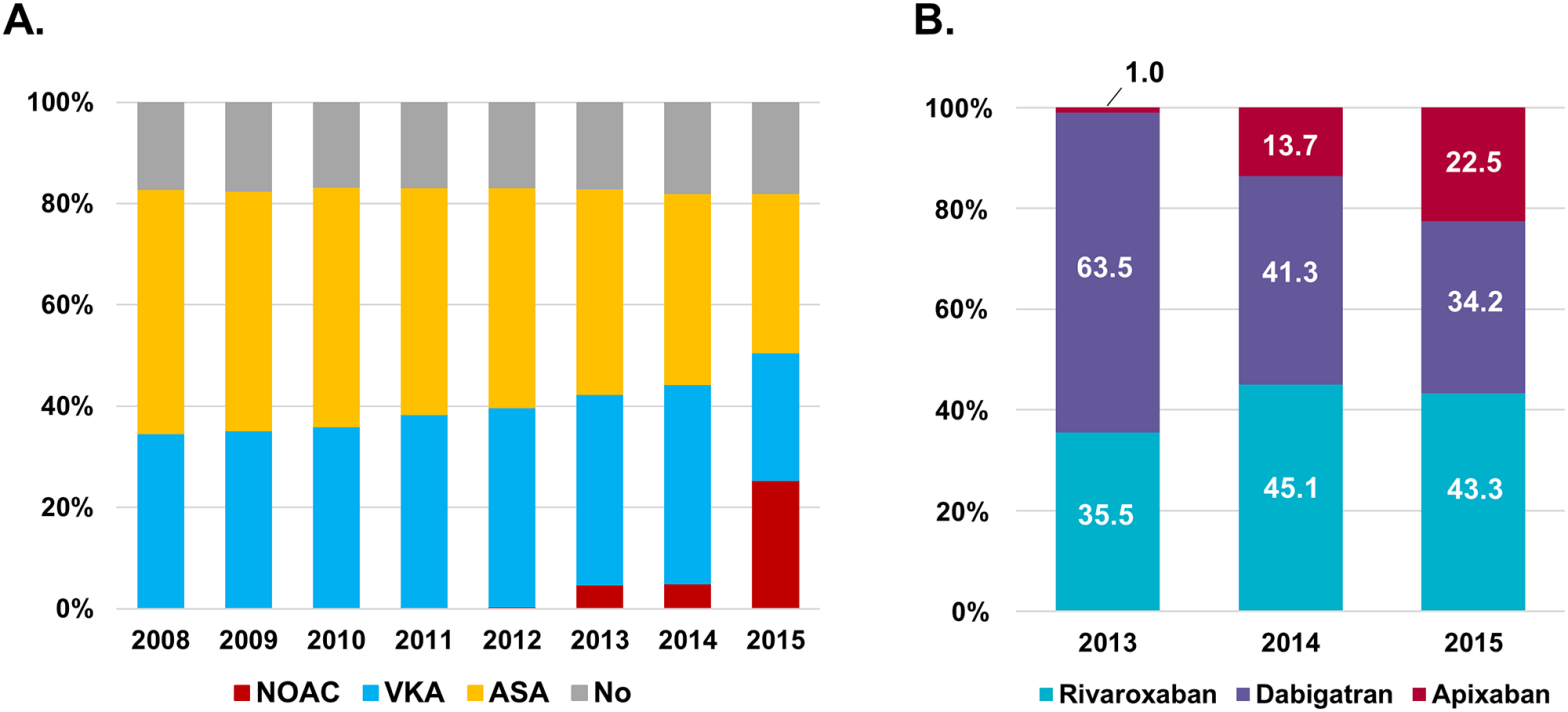
Temporal trends of antithrombotic therapy prescription. A. Temporal trends of antithrombotic therapy prescription in patients with CHA_2_DS_2_-VASc score ≥ 2. B. Distribution of three NOACs use since 2013 in patients with CHA_2_DS_2_-VASc score ≥ 2. Abbreviation: ASA, aspirin; NOAC, non-vitamin K oral anticoagulants; VKA, vitamin K antagonists.

A similar pattern of prescription of antithrombotic therapy was observed in patients with CHA_2_DS_2_-VASc score ≥ 2 (Fig 1A). The proportion of patients who received antithrombotic therapy (OAC or aspirin) was higher (approx. 82%) than the total study population. Approximately 17% of patients were prescribed no antithrombotic therapy throughout the study period. Aspirin prescription consistently decreased from 48.2% to 31.5%, while OAC prescription increased from 34.7% to 50.6% between 2008 and 2015 (p for trend < 0.001). After the limited approval of NOACs in 2013, 37.6% of patients received VKAs and 4.8% received NOACs. NOAC prescription increased with wider approval of NOACs in 2015 (25.4% of total patients with CHA_2_DS_2_-VASc score ≥ 2 and 50.2% of OAC prescriptions). Patient distribution by NOACs was similar within the total population (Fig 1B).

### Differences of baseline characteristics according to antithrombotic therapy in patients with CHA_2_DS_2_-VASc score ≥ 2 in 2015

Table 1 shows baseline characteristics for patients with CHA_2_DS_2_-VASc score ≥ 2 categorized according to the type of antithrombotic treatment. Patients receiving aspirin monotherapy were younger than patients receiving no therapy or OAC treatment, while women were more prevalent in the no therapy group. The prevalence of comorbidities did not show consistent trends.

As expected, older age, prior stroke/TIA/TE, diabetes and heart failure were more prevalent in the OAC group. Vascular disease, including prior MI and PAD, was more common in patients with aspirin monotherapy. Prior ICH was less common in the aspirin and OAC groups than in patients with no antithrombotic therapy.

Compared to patients with no OAC therapy, the OAC group had higher CHA_2_DS_2_-VASc score. In the OAC group, patients receiving NOACs were generally older and with a higher prevalence of prior stroke/TIA/TE and higher CHA_2_DS_2_-VASc score than patients receiving VKA. In addition, patients who had prior ICH were more common in the NOAC group.

### Comparison between men and women with CHA_2_DS_2_-VASc score ≥ 2

**Fig 2:**
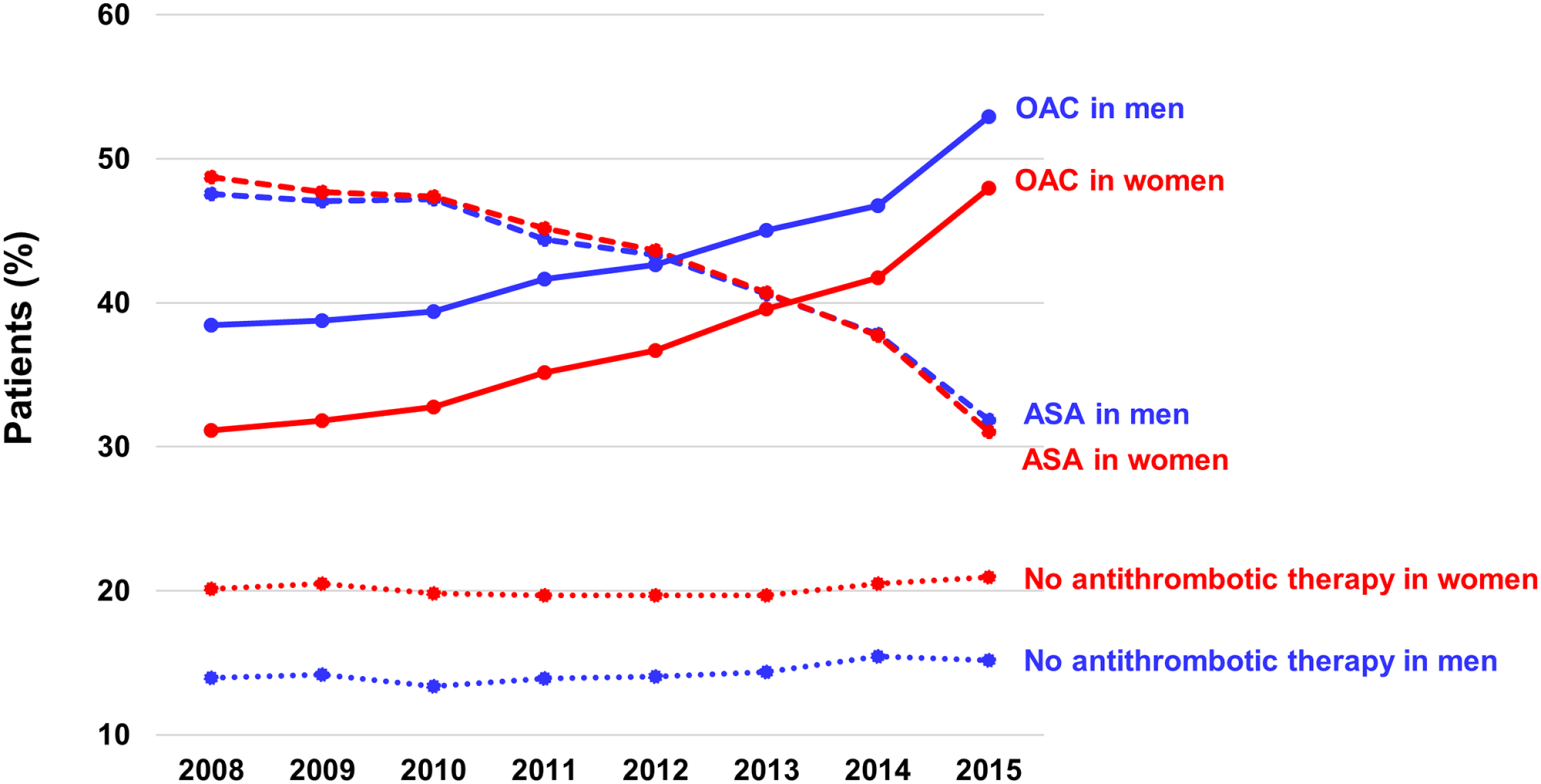
Comparison of temporal trends for each antithrombotic therapy strategy between men and women. Abbreviation: ASA, aspirin; NOAC, non-vitamin K oral anticoagulants.

Approximately 14–15% of men and 20–21% of women did not receive any antithrombotic agent for stroke prevention (S2A and S2B Fig). Aspirin prescription showed a gradual decrease in both sexes throughout the study period, and no significant sex differences. Women were more likely to be undertreated with OAC during the study period (p < 0.001), but the difference between men and women regarding the prevalence of OAC prescription has gradually decreased over time (from 7.3% in 2008 to 4.9% in 2015) (Fig 2).

### Men with 1 additional stroke risk factor (CHA_2_DS_2_-VASc score = 1)

**Fig 3:**
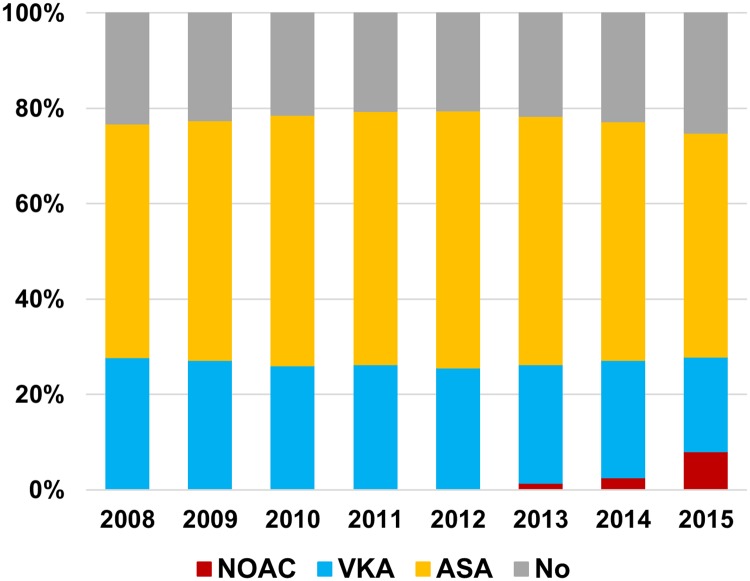
Temporal trends of antithrombotic therapy in men with CHA_2_DS_2_-VASc score of 1. Abbreviation: ASA, aspirin; NOAC, non-vitamin K oral anticoagulants; VKA, vitamin K antagonists.

Nearly one-half of these patients received aspirin during the study period. Aspirin prescription increased from 2008 to 2012 (49.0%% in 2008 and 53.9% in 2012), then decreased from 2012 to 2015 (46.9% in 2015). OAC prescription decreased between 2008 and 2011 (27.8% in 2008 and 26.3% in 2011), then slightly increased (28.0% in 2015). Almost one third of the OAC patients in 2015 received NOACs (Fig 3).

### Factors associated with OAC underuse

**Fig 4:**
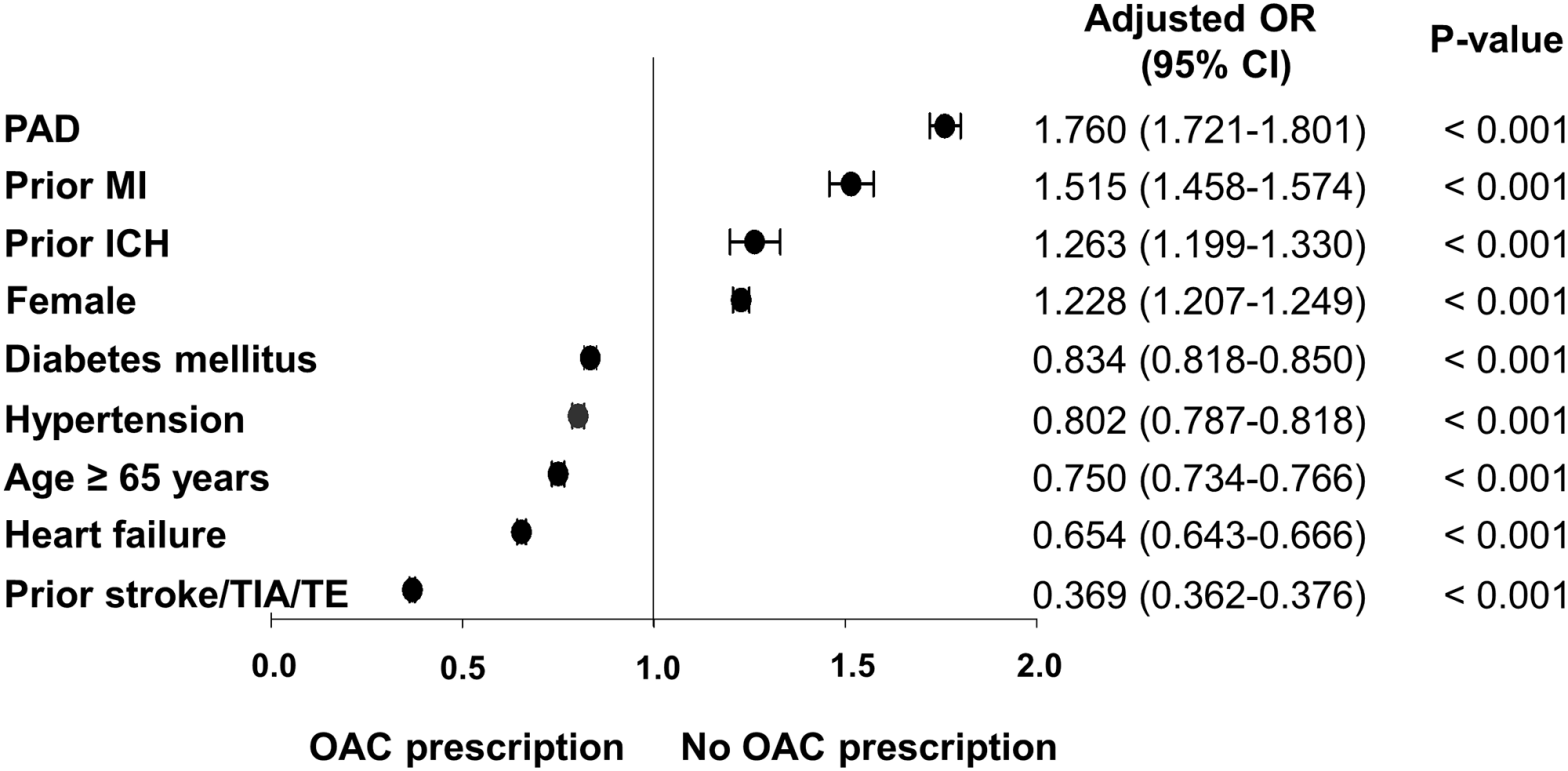
Factors associated with OAC underuse. Abbreviation: CI, confidence interval; ICH, intracranial hemorrhage; MI, myocardial infarction; OR, odds ratio; PAD, peripheral artery disease; TIA, transient ischemic attack; TE, systemic thromboembolism.

On multivariate logistic regression analysis, factors associated with OAC prescription pattern were old age, male, history of prior stroke/TIA/TE, and comorbidities (hypertension, diabetes, and heart failure), whereas female, vascular disease including prior MI and presence of PAD, and prior ICH were associated with OAC non-prescription (Fig 4).

## Discussion

We found significant temporal changes in the prescription patterns of antithrombotic therapy over 8 years in the entire Korean AF population. Our principal findings are as follows: (1) during the last 8 years, OAC prescription has gradually increased, especially after NOAC approval in 2013. In 2015, 46% of total population with AF, and 51% of patients with AF and CHA_2_DS_2_-VASc score ≥ 2, were receiving OAC treatment, and this proportion constantly increased over the study period; (2) aspirin prescription has gradually decreased in both the total population and the high stroke risk population; and (3) approximately 20% of total population with AF, and 17% of patients with AF and CHA_2_DS_2_-VASc score ≥ 2, were not receiving any antithrombotic therapy, and this proportion did not change over the study period. To the best of our knowledge, this is one of the first reports to describe the temporal trends in antithrombotic therapy in a nationwide Asian cohort, in the NOAC era.

### Changing patterns of antithrombotic therapy in patients with AF in the recent decade

Several studies have reported OAC underuse in patients with AF. The prescription rate of OAC varies widely depending on countries and study populations, ranging from 15% to 80% [3,4]. The Global Anticoagulant Registry in the FIELD-Atrial Fibrillation (GARFIELD-AF) reported a temporal change of prescription patterns of antithrombotic therapy in patients with newly diagnosed AF based on the comparisons over sequential cohorts between 2010 and 2015 [12]. OAC prescription rates in GARFIELD-AF increased from 57.4% to 71.1%, and this increase was largely driven by NOAC prescription. Similarly, data from the Global Registry on Long-Term Oral Antithrombotic Treatment in Patients with Atrial Fibrillation (GLORIA-AF) Phase 2 also showed an increase of OAC use in the era of NOACs [14]. Compared to the pre-NOACs era (Phase 1), the proportion of patients using OACs has markedly increased (from 64% to 80%, with NOACs proportionally greater (approx. 60%) than VKA overall), although regional differences were evident. A similar trend was observed in OAC treatment patterns in Danish nationwide registries from 2005 to 2015 [13].

In the Asian population, a higher proportion of patients with AF do not receive OAC for stroke prevention when compared to the European population, whether in the era of pre- or post-NOACs [14,24]. In GLORIA-AF, OAC use was 55.2% of the total Asian population with AF where about half of OAC users received NOACs [14]. Approximately 23.7% patients were prescribed aspirin, and 20% of patients did not receive any antithrombotic therapy. The antithrombotic therapy in our nationwide cohort study in 2015 shows a similar distribution with Asian regional data from GLORIA-AF.

Few reports on OAC use for stroke prevention are available for the Korean AF population. Lee et al. reported OAC underuse in patients with AF and CHADS_2_ score ≥ 2 and ATRIA bleeding risk score ≤ 4 using the National Patients Sample database of 2009 (n = 8,475) [5]. OAC use and antithrombotic therapy, including OAC and aspirin use, were estimated at 36.0% and 79.6%, respectively. Indeed, the proportions of OAC and overall antithrombotic therapy was similar to our results in the same year (2009) when VKAs were the only available OAC. More recently, OAC underutilization in the early era of NOACs has been reported [25]. In patients with AF from 2011 to 2014 from Aged Patient Sample, the proportion of OAC use in patients with CHA_2_DS_2_-VASc score ≥ 2 and ATRIA bleeding risk score ≤ 4 found that OAC use increased from 32% to 37.5% during the study period; however, the majority of OAC patients still received VKAs rather than NOACs. As the reimbursement criteria was modified in 2015, prescription patterns of OAC therapy changed significantly, as seen in our study.

### Real world practice compared to current guidelines

The decrease in aspirin use and increase in OAC use reflects major changes in the current guidelines in real world practice [7,10,11]. Reflecting the pivotal major trials and updated guidelines in other societies, the Koreans guidelines recommended NOACs or warfarin in patients with CHA_2_DS_2_-VASc score ≥ 1 [23]. Although OACs were still underused in 2015, prescription patterns for stroke prevention in patients with AF have changed according to the guidelines. Using the CHA_2_DS_2_-VASc score for stroke risk assessment would increase the number of patients at risk of stroke who would need OAC treatment by 17.5%, compared to previous guideline using CHADS_2_ scores [26]. In the current era of NOACs, physicians might initiate OAC treatment earlier and more easily using NOACs without any concern about frequent monitoring and dose adjustment. Nevertheless, the proportion of patients without any antithrombotic therapy did not change over time despite the many changes in guidelines and introduction of NOACs.

### Factors associated with OAC underuse

We found three factors, i.e. female gender, vascular disease, and prior ICH, were associated with non-use of OAC therapy in the NOAC era. Previous studies have reported on the underuse of OAC therapy in female patients with AF in accordance with our results [3,5,13,25]. The reason of OAC underuse in females was not fully understood, but in the VKA era, female gender was a risk factor for poorer anticoagulation control as assessed by time in the therapeutic range of the INR [27]. Women might have poorer access to medical care, which could influence the prescription of warfarin that needs frequent monitoring. Another possible explanation might be the difference in the age at the time of their first stroke between male and female patients. Females were generally older at the time of their first stroke [28]. It would be possible that women who started antithrombotic therapy could be older than male patients, and the number of population who were considered OAC treatment for secondary prevention would be smaller in females than in males especially at younger age. We had performed interaction analysis between age and gender on OAC prescription, and found a significant interaction between two factors (p-interaction < 0.001). Although we did not show the associated factor with OAC prescription stratified by age group with each gender or incident of stroke, we adjusted both gender and age as variables on multivariable logistic regression analysis for OAC prescription. Both age and gender affected the OAC prescription patterns. In concordance with our study results, the Denmark nationwide study reported that females were less likely to be initiated OAC compared with males, even though females are at increased risk of stroke [13]. Although the reasons behind OAC underuse in females is not well understood, the introduction of CHA_2_DS_2_-VASc score which counts female gender as a risk factor might affect the increased OAC use in females [13]. In the present study, gender differences in OAC use have decreased, especially after the introduction of CHA_2_DS_2_-VASc score and the availability of NOACs (Fig 2).

While known risk factors of stroke such as hypertension, diabetes, heart failure and prior stroke/TIA/TE were associated with OAC use, vascular disease, including prior MI and PAD, showed an association with no OAC prescription. Previous studies reported that the presence of vascular disease increased the OAC underuse [13,25]. Physicians’ underperception of vascular disease as an independent risk factor of stroke in AF patients and concomitant use of one or more anti-platelet agents in population with vascular disease could be possible reasons for non-prescription [29–31]. Concomitant use of anti-platelet agents and OAC would increase the risk of bleeding. This could be one of the reasons why OAC would not be prescribed in patients with anti-platelet agents, leading to underuse of OAC in this population.

### OAC underuse in men with 1 additional stroke risk

The use of OAC in patients with CHA_2_DS_2_-VASc score of 1 is controversial. The 2014 AHA/ACC/HRS guidelines state that no therapy, aspirin or OACs, might be considered for patients with a CHA_2_DS_2_-VASc score of 1 as a Class IIb recommendation [10], but this includes low risk females with no other risk factors who score 1 point by virtue of gender. In the 2016 ESC guideline, men with a CHA_2_DS_2_-VASc score of 1 and women with a CHA_2_DS_2_-VASc score of 2 should be considered for OAC therapy as a Class IIa recommendation [11,32]. In the 2015 Korean AF guidelines, patients with a CHA_2_DS_2_-VASc score of 1, OAC therapy should be preferentially considered, but depending on bleeding risk or patient preferences, antiplatelet therapy or no therapy could be permitted [23]. In current Korean medical insurance imbursement, NOAC prescription in men with a CHA_2_DS_2_-VASc score of 1 is not covered, which might limit the usage of NOAC in this population. We found that more than 75% of men with a CHA_2_DS_2_-VASc score of 1 were undertreated with OAC therapy. In our previous study, we reported that men with a CHA_2_DS_2_-VASc score of 1 were still at high stroke risk, at 1.47% per 100 person-years [21]. Based on the Taiwan nationwide data, the annual stroke rate was 2.75% in men with AF and CHA_2_DS_2_-VASc score of 1 and 2.55% in females with score 2, namely patients with one non-gender related stroke risk factor [33]. Nevertheless, stroke risk varies from 1.96%/year to 3.50%/year depending on the particular additional stroke risk factor, with age 65 to 74 years being the strongest driver of stroke risk (3.5%/year). In a recent meta-analysis, the estimated ischemic stroke risk was 1.61%/year in patients with CHA_2_DS_2_-VASc score of 1 [34], but again this includes low risk females who score 1 point by virtue of gender. Nevertheless, the population with one non-gender related stroke risk factor might be recommended NOACs rather than VKAs for stroke prevention, based on the positive net-benefit of treating such patients [35–37].

### Study limitations and strengths

A major limitation of this study was its retrospective observational character based on claims data from the NHIS database. The claims database did not provide patient-level clinical data that might provide information about OAC contraindications, such as formal bleeding risk score assessment. In addition, inadequate anticoagulation due to poor drug adherence or longitudinal data about interruption of antithrombotic therapy were not reflected in this study. Interruption of antithrombotic agents is not uncommon in real world practice. It is possible that AF patients without prescription of OAC might have received antithrombotic therapy in previous year, but interrupted with some reasons. Unfortunately, we could not analyze the information regarding the patients’ previous medication history including antithrombotic therapy due to our study design. We focused on analyzing the trends of OAC prescription patterns using cross-sectional prescription data. Also, inappropriate control of INR in patients using VKAs were not considered in this study.

Despite these limitations, this study includes unique descriptions of longitudinal data from the entire Korean AF population rather than data from selected or registered patients from trials, specific insurance claim providers or reimbursed sponsored registries. Therefore, our findings reflect the real-world clinical practice pattern of antithrombotic therapy at a nationwide scale.

## Conclusions

Between 2008 and 2015, a greater proportion of AF patients received OAC treatment with increasing NOAC prescription trends in the recent 3 years, and aspirin monotherapy has decreased substantially. A substantial proportion (approx. 50%) of Korean patients with AF still remain undertreated, being unaffected by the recent guideline updates or NOAC use. Further efforts to implement guidelines for stroke prevention are necessary to improve the outcomes of Asian patients with AF.

## Supporting information

S1 Fig Temporal trends of antithrombotic therapy prescription in total study population. A. Temporal trends of antithrombotic therapy prescription. B. Distribution of three NOACs use since 2013. Abbreviation: ASA, aspirin; NOAC, non-vitamin K oral anticoagulants; VKA, vitamin K antagonists.
(TIF)

S2 Fig Comparison of antithrombotic therapy between men and women. A. Temporal trends of antithrombotic therapy in men with CHA_2_DS_2_-VASc score ≥ 2. B. Temporal trends of antithrombotic therapy in women with CHA_2_DS_2_-VASc score ≥ 2. Abbreviation: ASA, aspirin; NOAC, non-vitamin K oral anticoagulants; VKA, vitamin K antagonists.
(TIF)

S1 Table Definition of comorbidities. (DOCX)

S2 Table Baseline characteristics of patients with CHA_2_DS_2_-VASc score 0,1 in 2015. (DOCX)
