# Supplementary figures and images for: Temporal trends of antithrombotic therapy for stroke prevention in Korean patients with non-valvular atrial fibrillation in the era of non-vitamin K antagonist oral anticoagulants: A nationwide population-based study
Source: PLoS One. 2017 Dec 20;12(12):e0189495. doi: 10.1371/journal.pone.0189495 (PMC5738023; doi:10.1371/journal.pone.0189495)

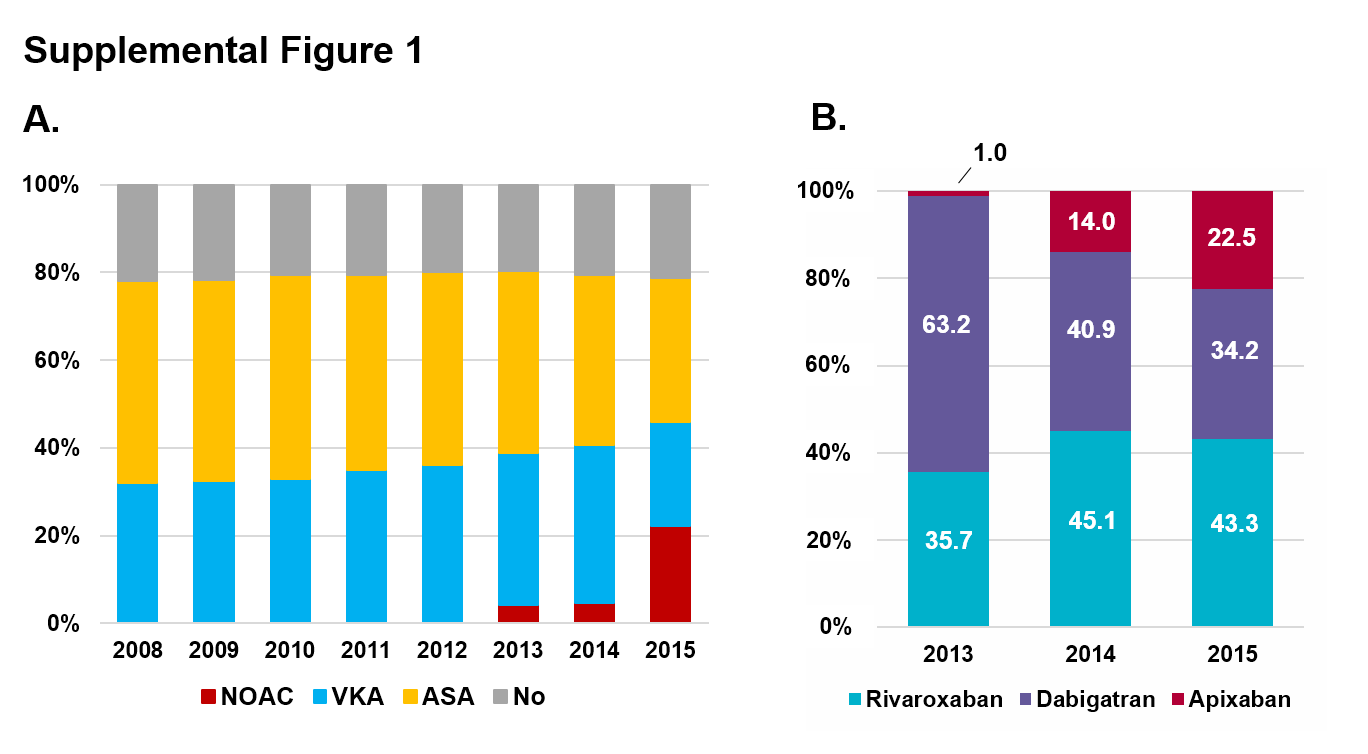

Supplement: S1 Fig — A. Temporal trends of antithrombotic therapy prescription. B. Distribution of three NOACs use since 2013. Abbreviation: ASA, aspirin; NOAC, non-vitamin K oral anticoagulants; VKA, vitamin K antagonists. (TIF) [file pone.0189495.s001.tif]

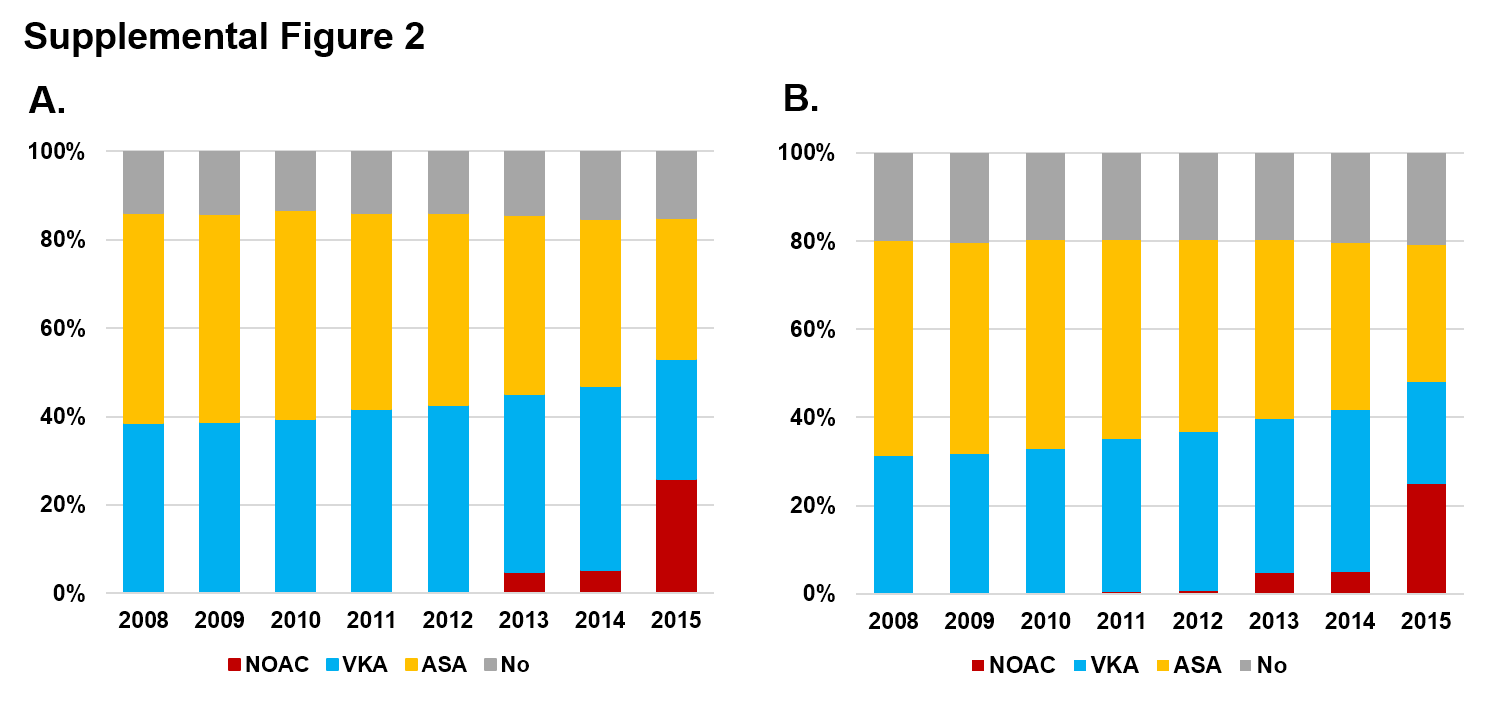

Supplement: S2 Fig — A. Temporal trends of antithrombotic therapy in men with CHA2DS2-VASc score ≥ 2. B. Temporal trends of antithrombotic therapy in women with CHA2DS2-VASc score ≥ 2. Abbreviation: ASA, aspirin; NOAC, non-vitamin K oral anticoagulants; VKA, vitamin K antagonists. (TIF) [file pone.0189495.s002.tif]
