# Supplementary material for: Temporal trends of antithrombotic therapy for stroke prevention in Korean patients with non-valvular atrial fibrillation in the era of non-vitamin K antagonist oral anticoagulants: A nationwide population-based study
Source: PLoS One. 2017 Dec 20;12(12):e0189495. doi: 10.1371/journal.pone.0189495 (PMC5738023; doi:10.1371/journal.pone.0189495)
Supplement: S1 Table — (DOCX) [file pone.0189495.s003.docx]

**S1 Table. Definition of comorbidities**

| Diagnosis | ICD-10-CM code and definition |
| --- | --- |
| Hypertension^a^ | I10-I13, I15; and minimum 1 prescription of anti-hypertensive drug (thiazide, loop diuretics, aldosterone antagonist, alpha-/beta-blocker, calcium-channel blocker, angiotensin-converting enzyme inhibitor, angiotensin II receptor blocker). |
| Diabetes mellitus^a^ | E11-E14; and minimum 1 prescription of anti-diabetic drugs (sulfonylureas, metformin, meglitinides, thiazolidinediones, dipeptidyl peptidase-4 inhibitors, α-glucosidase inhibitors and insulin). |
| Heart failure | I50 |
| Prior stroke | I63, I64 |
| Prior transient ischemic attack | G458, G459 |
| Prior systemic thromboembolism | I74 |
| Vascular disease |  |
| Prior myocardial infarction | I21, I22 |
| Peripheral artery disease | I70, I73 |
| Prior intracranial hemorrhage^b^ | I60-I62, I06.4-6 |

All variables except hypertension, diabetes mellitus and intracranial hemorrhage were defined when patients had one or more diagnoses during hospitalization or at outpatient clinic.

^a^ Hypertension and diabetes mellitus were identified when patients had ≥1 diagnoses during hospitalization or ≥2 diagnoses at outpatient clinic for preventing overestimation of diagnosis.

^b^ Intracranial hemorrhage was defined when patients had ≥1 diagnoses during hospitalization or received ≥1 pack of red blood cell transfusion.
