# Supplementary material for: Temporal trends of antithrombotic therapy for stroke prevention in Korean patients with non-valvular atrial fibrillation in the era of non-vitamin K antagonist oral anticoagulants: A nationwide population-based study
Source: PLoS One. 2017 Dec 20;12(12):e0189495. doi: 10.1371/journal.pone.0189495 (PMC5738023; doi:10.1371/journal.pone.0189495)
Supplement: S2 Table — (DOCX) [file pone.0189495.s004.docx]

**S2 Table. Baseline characteristics of patients with CHA_2_DS_2_-VASc score 0, 1 in 2015 (n = 45,914)**

|  | No OAC, total  (n=35,691) | No therapy  (n = 17,307) | Aspirin  (n = 18,384) | OAC, total  (n = 10,819) | VKA  (n = 7,796) | NOAC  (n = 3,023) |
| --- | --- | --- | --- | --- | --- | --- |
| Age (mean ± SD) | 53.7±11.0 | 50.6±12.3 | 56.6±8.6 | 56.9±9.6 | 56.7±9.6 | 57.5±9.6 |
| Age 65-74, n (%) | 3,790 (10.4) | 1,478 (8.5) | 2,312 (12.6) | 1,766 (16.3) | 1,158 (14.9) | 608 (20.1) |
| Age ≥ 75 years, n (%) | 0 (0) | 0 (0) | 0 (0) | 0 (0) | 0 (0) | 0 (0) |
| Female, n (%) | 6,386 (17.9) | 4,746 (27.4) | 1,640 (8.9) | 1,201 (11.1) | 933 (12.0) | 268 (8.9) |
| Hypertension, n (%) | 10,046 (28.2) | 3,115 (18.0) | 6,931 (37.7) | 3,603 (33.3) | 2,644 (33.9) | 960 (31.8) |
| Diabetes mellitus, n (%) | 1,210 (3.4) | 566 (3.3) | 644 (3.5) | 400 (3.7) | 296 (3.8) | 104 (3.4) |
| Heart failure, n (%) | 2,568 (7.2) | 884 (5.1) | 1.684 (9.2) | 1,280 (11.8) | 923 (11.8) | 357 (11.8) |
| Prior stroke/TIA/TE, n (%) | 0 (0) | 0 (0) | 0 (0) | 0 (0) | 0 (0) | 0 (0) |
| Vascular disease, n (%) | 1,001 (2.8) | 449 (2.6) | 552 (3.0) | 181 (1.7) | 132 (1.7) | 49 (1.6) |
| Prior MI, n (%) | 250 (0.7) | 128 (0.7) | 122 (0.7) | 45 (0.4) | 30 (0.4) | 15 (0.5) |
| PAD, n (%) | 771 (2.2) | 331 (1.9) | 440 (2.4) | 142 (1.3) | 106 (1.4) | 36 (1.2) |
| Prior ICH | 551 (1.5) | 391 (2.3) | 160 (0.9) | 128 (1.2) | 90 (1.2) | 38 (1.3) |
| CHA_2_DS_2_-VASc score (mean ± SD) | 0.7±0.5 | 0.7±0.5 | 0.8±0.4 | 0.8±0.4 | 0.8±0.4 | 0.8±0.4 |
| CHA_2_DS_2_-VASc score, n (%) |  |  |  |  |  |  |
| Score = 0 | 10,678 (29.9) | 6,063 (35.0) | 4,615 (25.1) | 2381 (22.0) | 1706 (21.9) | 675 (22.3) |
| Score = 1 | 25,013 (70.1) | 11,244 (65.0) | 13,769 (74.9) | 8,438 (78.0) | 6,090 (78.1) | 2,348 (77.7) |
